# The role of monoamine oxidase A in HPV-16 E7-induced epithelial-mesenchymal transition and HIF-1α protein accumulation in non-small cell lung cancer cells

**DOI:** 10.7150/ijbs.46966

**Published:** 2020-08-01

**Authors:** Bingyu Huang, Zhiyuan Zhou, Jiao Liu, Xin Wu, Xiangyong Li, Qiang He, Peihua Zhang, Xudong Tang

**Affiliations:** 1 Institute of Biochemistry and Molecular Biology, Collaborative innovation center for antitumor active substance research and development, Guangdong Provincial Key Laboratory of Medical Molecular Diagnostics, Guangdong Medical University, Zhanjiang 524023, P.R. China.; 2 Marine Medical Research Institute of Guangdong Zhanjiang, Guangdong Key Laboratory for Research and Development of Natural Drugs, Guangdong Medical University, Zhanjiang 524023, P.R. China.; 3 Institute of Plastic Surgery, Affiliated Hospital of Guangdong Medical University, Zhanjiang 524001, P.R. China.

**Keywords:** epithelial-mesenchymal transition (EMT), human papillomavirus (HPV), hypoxia-inducible factor-1α (HIF-1α), monoamine oxidases A (MAOA), non-small cell lung cancer (NSCLC)

## Abstract

Our previous studies have found that human papillomavirus (HPV)-16 E7 oncoprotein promotes epithelial-mesenchymal transition (EMT) and hypoxia-inducible factor-1α (HIF-1α) protein accumulation in non-small cell lung cancer (NSCLC) cells and monoamine oxidase A (MAOA) is highly expressed in NSCLC tissues. Here, we further explored the role of MAOA in HPV-16 E7-induced EMT and HIF-1α protein accumulation in A549 and NCI-H460 NSCLC cells. Our results showed that HPV-16 E7 enhanced MAOA expression in NSCLC cells. Additionally, MAOA knockout inhibited HPV-16 E7-induced migration, invasion, and EMT, and significantly reduced HPV-16 E7-induced ROS generation and HIF-1α protein accumulation *via* promoting its degradation. Furthermore, MAOA knockout suppressed HPV-16 E7-induced ERK1/2 activation. *In vivo*, MAOA knockout inhibited tumor growth, metastasis, and the expression of EMT-related markers and HIF-1α proteins induced by HPV-16 E7 in NCI-H460 NSCLC subcutaneous xenograft and *in situ* intrapulmonary models of nude mice. Taken together, our findings provide evidence that MAOA plays a key role in EMT and HIF-1α protein accumulation induced by HPV-16 E7 in NSCLC cells, suggesting that MAOA may be a potential therapeutic target for HPV-related NSCLC.

## Introduction

Lung cancer is the most common cause of cancer mortality worldwide, accounting for 18.4% of cancer-related deaths worldwide 1. Lung cancer can be classified into small cell lung cancer (SCLC) and non-small cell lung cancer (NSCLC), and NSCLC accounts for about 80% of primary lung cancer 2, 3 with a low 5-year survival rate (7.0%) and median survival time (5 months) 4, 5. The risk factors for NSCLC include smoking, exposure to occupational and environmental carcinogens such as asbestos, arsenic, radon, and infection such as human papillomavirus (HPV) infection 6-9.

HPV is a double-stranded and non-enveloped DNA virus. Syrjanen first proposed a hypothesis that HPV can induce the occurrence of squamous cell lung cancer in 1979 10. Over the past 30 years, many subtypes of HPV infections have been found in lung cancer tissues, of which high-risk HPV-16 had the highest infection rate 11, 12. HPV-16 DNA and HPV-16 E6 and E7 oncoproteins were detected in lung cancer tissues and were considered as the potential causes of NSCLC 13, 14. However, the mechanisms by which HPV-16 oncoproteins promote NSCLC progression have not yet been fully elucidated.

Monoamine oxidase A (MAOA), a mitochondria-bound enzyme, can catalyze the degradation of monoamine neurotransmitters and dietary amines by oxidative deamination 15. Previous studies have shown that MAOA defect is closely related to impulsive aggressive behavior and negative emotions such as anxiety, depression, and anger 16, 17. Recently, accumulating evidence has demonstrated that MAOA plays an important role in cancer progression 15, 18-22. Our previous study has also found that MAOA protein and mRNA levels in NSCLC tissues are higher than those in the matched non-tumor adjacent lung tissues, and MAOA expression in NSCLC tissues is closely related to epithelial-mesenchymal transition (EMT) 23. However, the role of MAOA in HPV-16 oncoprotein-induced NSCLC has not been reported.

The poor prognosis in NSCLC is associated with the EMT, a key process that drives cancer metastasis 24. A growing body of evidence has demonstrated that EMT is closely related to tumorigenesis, invasion, distant metastasis, chemotherapy resistance, etc 25-28. The main features of EMT are the loss of epithelial cell phenotype such as the decrease of E-cadherin expression, the gain of mesenchymal cell phenotype such as the increase of N-cadherin and Vimentin expression, and the induction of EMT-related transcription factors (EMT-TFs) such as Snail1, Slug (Snail2), and Twist1 12, 29, 30. Yue et al. found that EMT promoted tumor invasion and migration abilities in NSCLC 31. Eo et al. also demonstrated that NSCLC cells with the lower EMT were more sensitive to tyrosine-kinase inhibitors than those with the higher EMT 32. These reports indicate that EMT mediates the progression of NSCLC. Interestingly, our previous study found that HPV-16 E7 oncoprotein enhanced EMT in NSCLC cells 33. However, the underlying molecular mechanisms of EMT enhanced by HPV-16 E7 oncoprotein in NSCLC cells still remain unclear.

Under normoxic conditions, prolyl hydroxylase enzymes (PHD) hydroxylates key proline residues of hypoxia-inducible factor-1α (HIF-1α), thereby allowing the von Hippel-Lindau protein to bind and target HIF-1α for proteasomal degradation. However, under hypoxic conditions, PHD activity is inhibited, resulting in the stabilization and translocation of HIF-1α to the nucleus 34. The hypoxic microenvironment is closely associated with cancer progression and metastasis. Recently, HIF-1α has been shown to up-regulate EMT-TFs and antagonize p53 35. Interestingly, our previous study demonstrated that overexpression of HPV-16 E7 oncoprotein enhanced HIF-1α protein accumulation and HIF-1α-dependent VEGF and IL-8 expression in NSCLC cells 36. However, the mechanisms by which HPV-16 E7 oncoprotein mediates the high expression of HIF-1α are not completely clear.

In this study, we found for the first time, to the best of our knowledge, that HPV-16 E7 oncoprotein promoted the expression of MAOA in NSCLC cells. Moreover, MAOA knockout inhibited EMT and HIF-1α protein accumulation induced by HPV-16 E7 oncoprotein in NSCLC cells. These results suggest that MAOA may be a potential therapeutic target for HPV-related NSCLC.

## Materials and Methods

### Reagents

Mouse anti-HPV-16 E7 antibody and rabbit anti‑human MAOA and MMP-2 antibodies were obtained from Abcam (Cambridge, UK). Mouse anti‑human Ki-67, rabbit anti‑human E‑cadherin, N-cadherin, Vimentin, Slug, Twist1, MMP-9, p-ERK1/2, ERK1/2, p-AKT, AKT, and β-actin monoclonal antibodies, and horseradish peroxidase‑conjugated secondary antibodies were from Cell Signaling Technology Inc. (Beverly, MA, USA). Mouse anti-human HIF-1α monoclonal antibody was purchased from BD Transduction Laboratories (San Diego, CA, USA). The RNA extraction kits (RNAprep Pure FFPE kits) were obtained from Tiangen Biotech Co., Ltd. (Beijing, China). The reverse transcription (RT) kit (PrimeScript™ RT reagent kits) and the qPCR analysis kit (SYBR Premix Ex Taq™ II) were purchased from Takara Biotechnology Co., Ltd. (Dalian, China). PD98059 was purchased from Cell Signaling Technology Inc. (Beverly, MA, USA). The human vascular endothelial growth factor (VEGF) ELISA kit was purchased from Boster Biological Technology Co.Ltd (Wuhan, China). The ROS assay kit was purchased from Beyotime Biotechnology Co. Ltd (Shanghai, China).

### Cell Lines and Cell Culture

A549 and NCI-H460 human NSCLC cell lines were purchased from American Type Culture Collection (Rockville, MD, USA) and Chinese Academy of Sciences Cell Bank of Type Culture Collection (Shanghai, China), respectively. All NSCLC cells were grown in RPMI-1640 media containing 10% fetal bovine serum, penicillin (100 U/ml), and streptomycin (100 μg/ml) at 37°C in a humidified condition with 5% CO_2_.

### Construction of stable HPV-16 E7-overexpressing cell lines

The GV287 lentivirus vector harboring HPV-16 E7 (GV287-HPV-16 E7) was constructed by Shanghai GK Gene Technology Co., Ltd. A549 and NCI-H460 NSCLC cells were infected with HPV-16 E7-lentivirus, and the stable HPV-16 E7- overexpressing A549 and NCI-H460 cell lines (abbreviation: 16 E7) were constructed. The empty vector-infected cells served as controls. The infected cells containing enhanced green fluorescent protein (EGFP) were sorted by flow cytometry. The expression of HPV-16 E7 oncoprotein in the infected cells was examined by Western blotting.

### Construction of stable cell lines with HPV-16 E7 overexpression and MAOA knockout

CRISPR/Cas9 technology was performed to knock out *MAOA* in the stable HPV-16 E7-overexpressing NSCLC cells to establish stable cell lines with HPV-16 E7 overexpression and MAOA knockout (abbreviation: 16 E7-MAOA KO). A lentivirus vector (U6-sgRNA-EFS-Cas9-P2A-Puro) was constructed by Shanghai Fubaiao Biotechnology Co., Ltd. To obtain 16 E7-MAOA KO cells, the infected cells were screened by incubation with 2 μg/ml of puromycin. The efficiency of MAOA knockout was analyzed by Western blotting.

### Enzyme-linked immunosorbent assay (ELISA)

The stable-infected cells (Empty vector, 16 E7, and 16 E7-MAOA KO) were cultured for 24 h, and the VEGF protein concentration in the conditional media was measured using a human VEGF ELISA kit (Boster Biological Technology Co.Ltd.) according to the manufacturer's instructions. The experiments were repeated in triplicate.

### Reactive oxygen species (ROS) detection

The stable-infected cells (Empty vector, 16 E7, and 16 E7-MAOA KO) were cultured for 16 h, followed by ROS analysis. The intracellular ROS level was detected by flow cytometry using a ROS assay kit (Beyotime Biotechnology Co. Ltd.) according to the manufacturer's instructions.

### Wound healing

Wound healing experiment was performed to quantify the MAOA effect on the motility of the stable-infected cells (Empty vector, 16 E7, and 16 E7-MAOA KO). The method was as described previously 37.

### Transwell Migration and invasion assays

The assays were performed using 24-well Transwell chambers (Corning Costar, Corning, NY) containing 8 μm pore size polycarbonate membranes with Matrigel (BD Biosciences, San Jose, CA) for invasion assay or without Matrigel for migration assay. The cells were starved overnight and inoculated into the upper chamber without serum. 16~24 h later, the passed cells under the chamber were fixed with 4 % formaldehyde, and then stained with 4 % crystal violet for 20 min. Under the microscope, 5 fields were randomly selected to calculate the number of the passed cells.

### RT-qPCR

Total RNA was extracted from Empty vector, 16 E7, and 16 E7-MAOA KO cells using TRIzol® reagent. The mRNA relative levels were determined using a RT kit and a qPCR kit (SYBR Green) according to the manufacturer's instructions (Tiangen Biotech). The sequences of the primers synthesized by Sangon Biotech (Shanghai, China) were as follows: *MAOA* forwards 5′-AGTGAGCGAACGGATAATGG-3′ and reverse 5′-TGTTCATGGTTCAG-CGTCTC-3′ [Genbank: NM_001270458.1]; *E-cadherin* forwards 5′-TTGCTACTGGAACAGGGACAC-3′ and reverse 5′-CCCGTGTGTTAG-TTCTGCTGT-3′ [Genbank: NM_004360.5]; *N-cadherin* forwards 5′-TTATCCTTGTGCTGATGTTTGTG-3′ and reverse 5′-TCTTCTTCTCCTCCACCTTCTTC-3′ [Genbank: NM_001792.5]; *Vimentin* forwards 5′-GAGAACTTTGCCGTTGAAGC-3′ and reverse 5′-TCCAGCAGCTTCCTGTAG-3′ [Genbank: NM_003380.5]; *Slug* forwards 5′-GAGCATACAGCCCCATCACT-3′ and reverse 5′-GGGTCTGAAAGCTT-GGACTG-3′ [Genbank: NM_003068.5]; *Twist1* forwards 5′-GTCCGCAG-TCTTACGAGGAG-3′ and reverse 5′-CCAGCTTGAGGGTCTGAATC-3′ [Genbank: NM_000474.4]; *β-actin* forwards 5′-TGACGTGGACATCCGCAAAG-3′ and reverse 5′-CTGGAAGGTGGACAGCGAGG-3′ [Genbank: NM_001101.5]. The RT conditions were as follows: 25°C for 10 min, 55°C for 30 min, and 85°C for 5 min. The qPCR conditions were as follows: 95°C for 5 min, followed by 40 cycles at 95°C for 10 s, 60°C for 20 s, and 72°C for 20 s. All relative mRNA levels were normalized to *β-actin*.

### Western Blotting

The cells were lysed on ice for 60 min with lysis buffer (Beyotime Biotechnology Corporation, Shanghai, China) and complete protease inhibitor cocktail (Sigma-Aldrich, St. Louis, MO, USA), and the supernatants were collected by centrifugation at 12,000×g for 10 min. The electrophoresis was performed on 10% SDS-PAGE gel and transferred to the polyvinylidene difluoride membrane. The membrane was blocked with 5% non-fat milk or BSA, and incubated with specific primary antibody and horseradish peroxidase-conjugated secondary antibody, respectively. Antigen-antibody complexes were visualized using enhanced chemiluminescence. The analysis of β-actin protein expression was used as an internal control.

### Animal experiments

The 4~5-week-old BALB/c nude mice were purchased from Beijing Vital River Laboratory Animal Technology Co., Ltd. (Beijing, China). The study was approved by Ethics Committee of Guangdong Medical University. The nude mice were maintained under a controlled temperature (20~26 °C), humidity (40~70%), and 12 h light/12 h dark cycle. The stable empty vector, 16 E7, and 16 E7-MAOA KO NCI-H460 cells (2×10^6^ cells/200 μl) were respectively injected subcutaneously into the left armpit of nude mice to establish the subcutaneous xenograft model, and the stable empty vector, 16 E7, and 16 E7-MAOA KO NCI-H460 cells (5×10^5^ cells/50 μl) were respectively mixed with Matrigel at a 1:1 ratio and the mixtures were respectively injected into the left lung of nude mouse to establish *in situ* intrapulmonary model. Both subcutaneous xenograft and *in situ* intrapulmonary models were classified into three groups (empty vector group, 16 E7 group, and 16 E7-MAOA KO group, 8 mice/group). The growth and diet of nude mice were observed, and the volume of subcutaneous tumors and the weight of nude mice were measured every three days. About 4 weeks later, all the nude mice were sacrificed. The tumors were weighed and fixed in 10% formalin.

### Immunohistochemistry

Immunohistochemical staining was performed on paraffin-embedded tissue specimens. The method was as described previously 23.

### Statistical Analysis

All data in this study were expressed as mean ± SD for at least three independent experiments. One-way ANOVA and *t* test were used as statistical analysis by GraphPad Prism 5.0 software. *P* < 0.05 was considered significant.

## Results

### HPV-16 E7 oncoprotein enhanced MAOA expression in NSCLC cells

**Figure 1:**
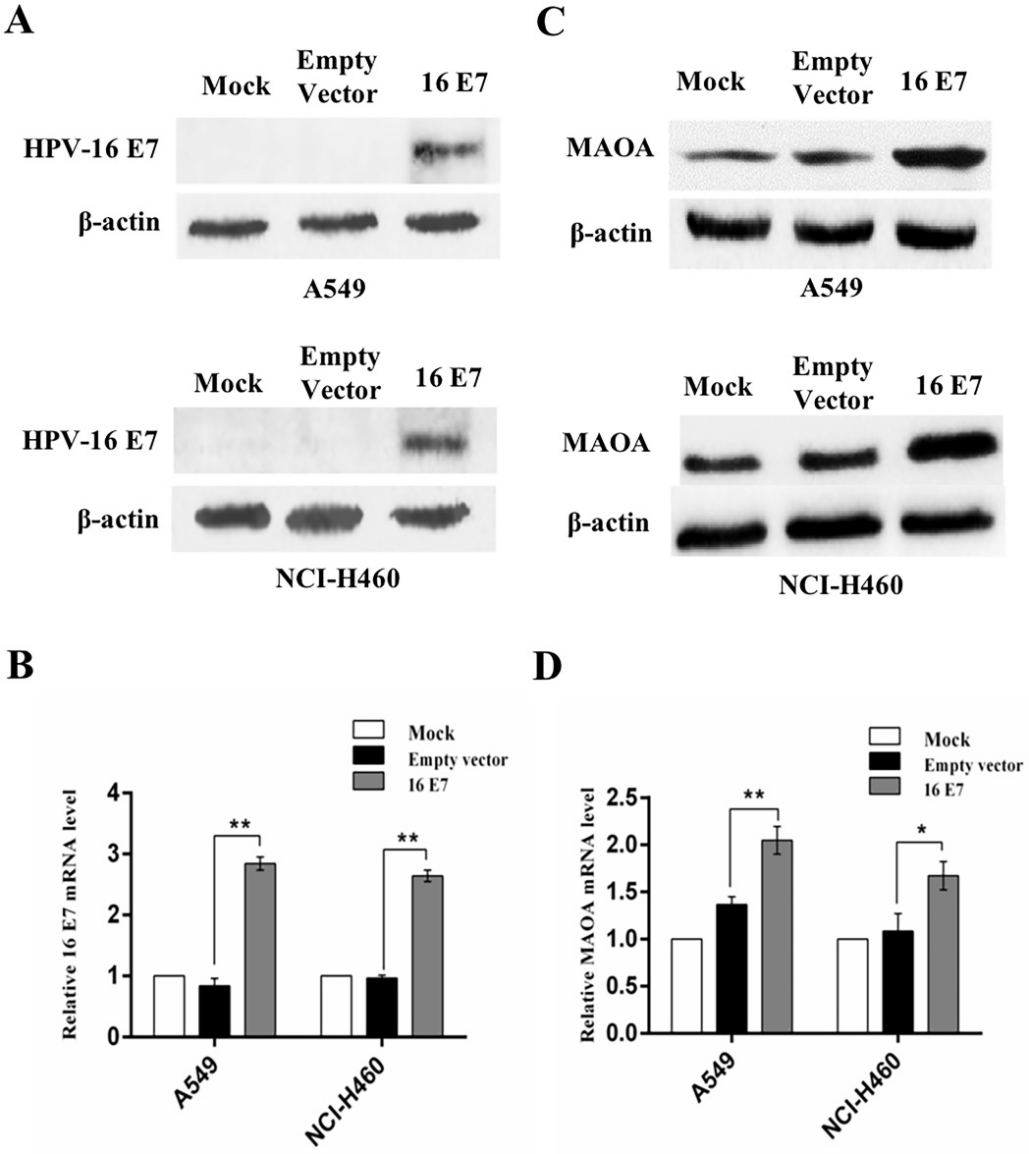
** Overexpression of HPV-16 E7 oncoprotein promoted the expression of MAOA in NSCLC cells. (A,B)** HPV-16 E7 protein (**A**) and mRNA (**B**) levels were determined by Western blotting and RT-qPCR, respectively. **(C,D)** MAOA protein (**C**) and mRNA (**D**) levels were determined by Western blotting and RT-qPCR, respectively. All data are expressed as mean±SD of three independent experiments. *P<0.05, **P<0.01.

Our previous study demonstrated that MAOA was highly expressed in human NSCLC tissues 23. However, to date, the effect of HPV-16 E7 on MAOA expression in NSCLC cells has not been reported. To address this question, we constructed the stable HPV-16 E7-overexpressing A549 and NCI-H460 cell lines (abbreviation: 16 E7). HPV-16 E7 protein and mRNA expression was confirmed in the infected cells (Figure 1A,B), indicating that the stable HPV-16 E7-overexpressing A549 and NCI-H460 cell lines (16 E7) were successfully constructed.

Next, we analyzed MAOA expression in the stable HPV-16 E7-overexpressing NSCLC cells (16 E7). Our results showed that the expression of MAOA protein was significantly stronger in HPV-16 E7-overexpressing cells than that in empty vector and mock infection controls (Figure 1C). Similar results were observed in *MAOA* mRNA level (Figure 1D). Taken together, our results demonstrated that HPV-16 E7 oncoprotein promoted MAOA expression at both protein and mRNA levels in A549 and NCI-H460 NSCLC cells.

### MAOA knockout inhibited HPV-16 E7-induced migration and invasion abilities of NSCLC cells

**Figure 2:**
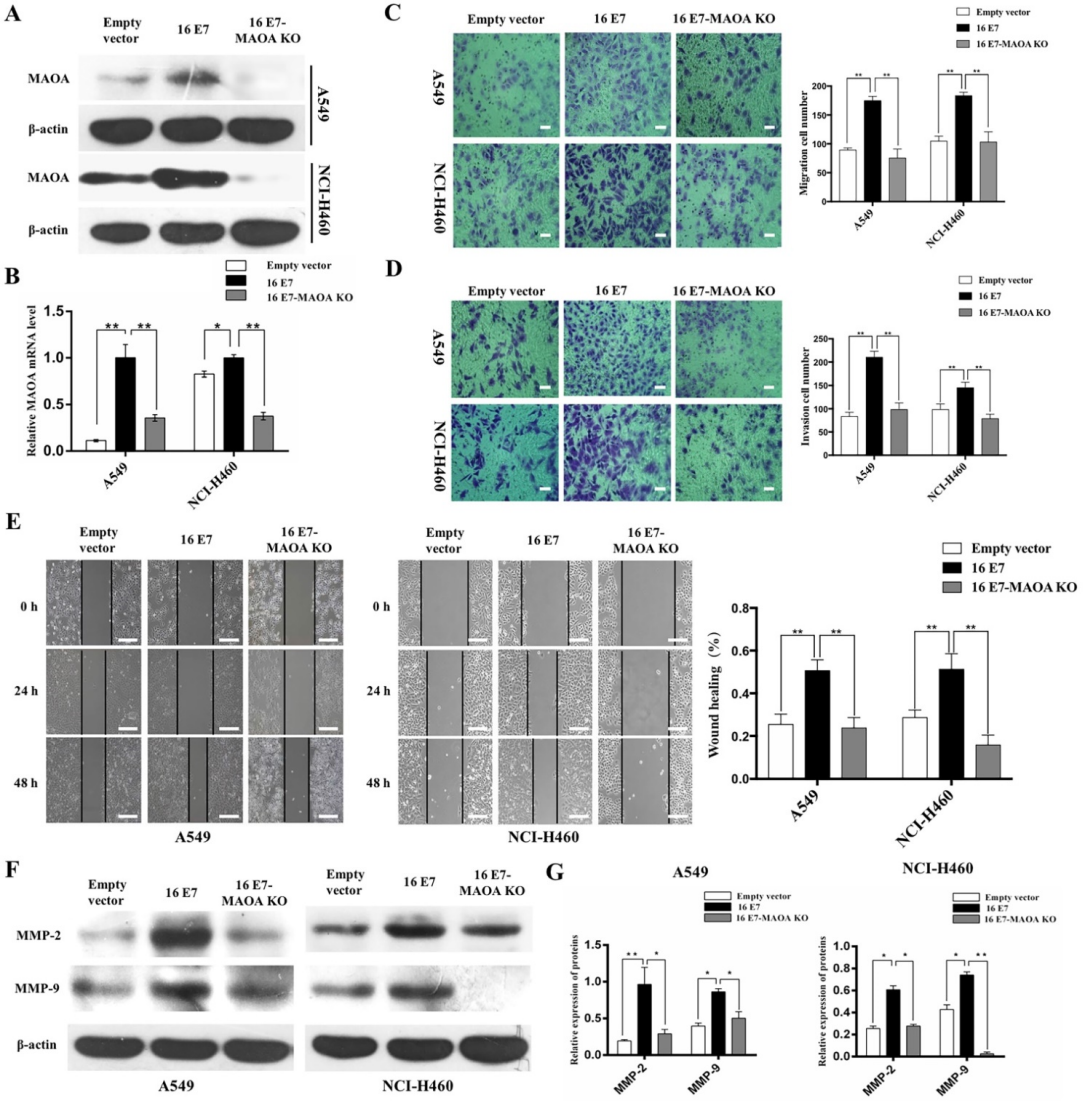
** MAOA knockout inhibited HPV-16 E7-induced migration and invasion abilities of NSCLC cells. (A, B)** The expression of MAOA protein (**A**) and mRNA (**B**) in stable A549 and NCI-H460 cells. **(C)** The results of transwell migration assay, scale bar =100 µm. **(D)** The results of transwell invasion assay, scale bar =100 µm. **(E)** The results of NSCLC cells motility by wound healing assay, scale bar =500 µm. (**F**) The expression of MMP-2 and MMP-9 in stable A549 and NCI-H460 cells. (**G**) The quantitative analysis for the expression of MMP-2 and MMP-9. All data are expressed as mean±SD of three independent experiments. *P<0.05, **P<0.01. Abbreviations: Empty vector, stable empty vector-infected cells; 16E7, stable HPV-16 E7-overexpressing cells; 16 E7-MAOA KO, stable cells with HPV-16 E7 overexpression and MAOA knockout.

Our previous studies demonstrated that HPV-16 E7 promoted the progression and development of NSCLC 12, 36. To study the role of MAOA in the progression and development of NSCLC promoted by HPV-16 E7, CRISPR/Cas9 technique was used to knock out *MAOA* in HPV-16 E7-overexpressing NSCLC cells (16 E7) to construct stable NSCLC cell lines with HPV-16 E7 overexpression and MAOA knockout (16 E7-MAOA KO). As expected, MAOA was knocked out in these cells (Figure 2A,B), indicating that the stable 16 E7-MAOA KO cells were successfully constructed.

Previous studies found that HPV-16 enhanced migration and invasion abilities of NSCLC cells 13, 38. To investigate the effect of MAOA on migration and invasion abilities promoted by HPV-16 E7, Transwell and wound healing assays were used to analyze the migration and invasion abilities of NSCLC cells. Our results showed that MAOA knockout significantly inhibited HPV-16 E7-induced migration (*P*<0.01, Figure 2C,E) and invasion (*P*<0.01, Figure 2D) abilities of A549 and NCI-H460 cells. Additionally, overexpression of HPV-16 E7 dramatically enhanced the expression of MMP-2 and MMP-9, whereas MAOA knockout inhibited this effect (*P*<0.01, Figure 2F,G). Collectively, our results indicated that MAOA might play a role in interfering with HPV-16 E7-induced migration and invasion abilities of NSCLC cells.

### MAOA knockout inhibited HPV-16 E7-induced EMT in NSCLC cells

**Figure 3:**
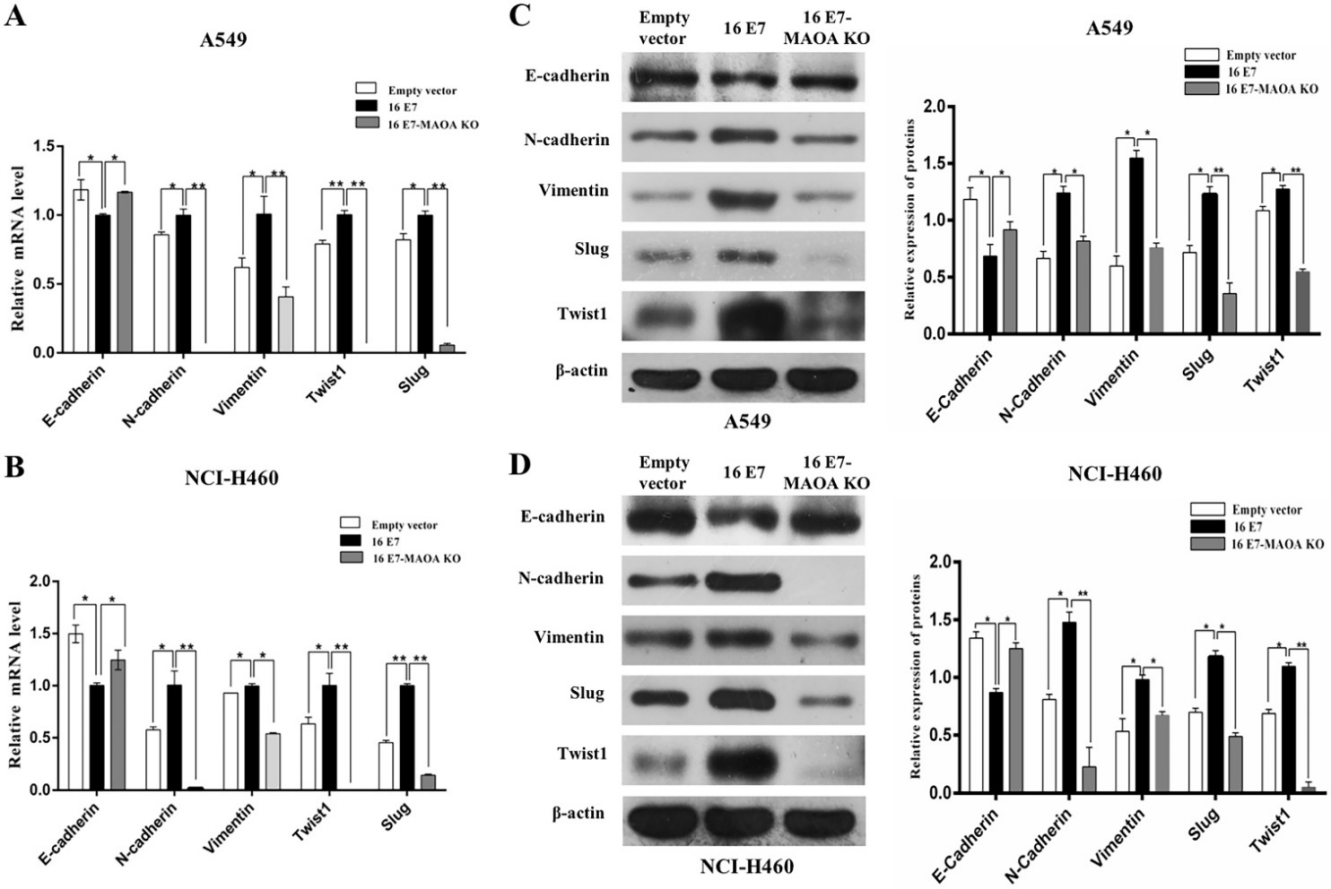
** MAOA knockout inhibited HPV-16 E7-induced EMT in NSCLC cells. (A,B)** RT-qPCR was performed to analyze the mRNA levels of *E-cadherin*, *N-cadherin*, *Vimentin*, *Slug*, and *Twist1* in stable A549 (**A**) and NCI-H460 (**B**) cells.** (C,D)** Western blotting was performed to analyze the protein levels of E-cadherin, N-cadherin, Vimentin, Slug, and Twist1 in stable A549 (**C**) and NCI-H460 (**D**) cells. All data are expressed as mean±SD of three independent experiments. *P < 0.05, **P < 0.01.

EMT, the first step of migration and invasion, promotes NSCLC tumorigenesis and progression 39. Interestingly, our previous study firstly demonstrated that HPV-16 E7 enhanced EMT, leading to NSCLC progression 12. Therefore, to further verify whether MAOA was involved in HPV-16 E7-induced EMT process in A549 and NCI-H460 cells, we investigated the mRNA levels of EMT-related epithelial marker (*E-cadherin*), mesenchymal markers (*Vimentin* and* N-cadherin*), and transcription factors (*Twist1* and *Slug*) by RT-qPCR. Our results showed that HPV-16 E7 down-regulated the *E-cadherin* mRNA level while up-regulated *Vimentin*, *N-cadherin*, *Twist1*, and *Slug* mRNA expression, but MAOA knockout inhibited these effects (Figure 3A,B). The similar results were observed in the expression of these proteins (Figure 3C,D). Collectively, these results suggested that MAOA knockout inhibited EMT induced by HPV-16 E7 oncoprotein in NSCLC cells.

### MAOA was involved in HPV-16 E7 -induced HIF-1α/VEGF signaling

**Figure 4:**
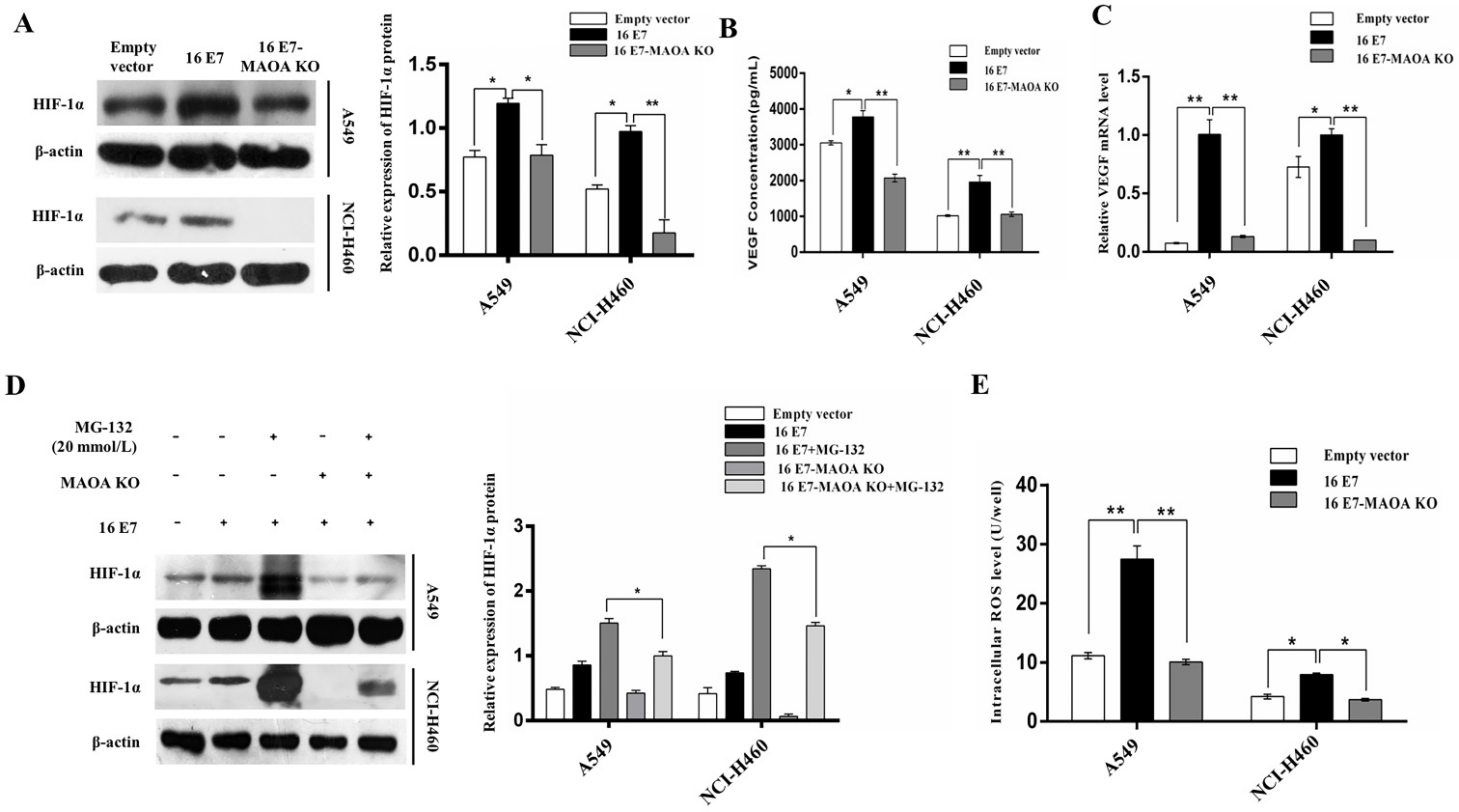
** MAOA knockout regulated HPV-16 E7-induced HIF-1α protein stability in NSCLC cells. (A)** HIF-1α protein expression was analyzed by Western blotting. **(B)** VEGF protein concentration in the conditional media derived from stable A549 and NCI-H460 cells was determined by ELISA. **(C)** RT-qPCR analysis of* VEGF* mRNA expression in stable A549 and NCI-H460 cells. **(D)** The stable A549 and NCI-H460 cells (16 E7 and 16 E7-MAOA KO) were treated with MG132 (20 mmol/L) for 24 h, followed by analysis of HIF-1α protein expression. **(E)** The intracellular ROS level was determined by flow cytometry. All data are expressed as mean±SD of three independent experiments. *P < 0.05, **P < 0.01.

Our previous study found that HPV-16 E7 oncoprotein enhanced HIF-1α/VEGF- mediated tumor angiogenesis in NSCLC cells 36. In this study, we further investigated the role MAOA in HPV-16 E7-induced HIF-1α accumulation under normoxic conditions. We demonstrated that HPV-16 E7-induced HIF-1α protein accumulation was significantly reduced by MAOA knockout (Figure 4A).

HIF-1α mediates VEGF expression in tumor microenvironment 40. In the present study, we measured the concentration of VEGF protein in the conditional media of NSCLC cells by ELISA. Our results showed that MAOA knockout significantly reduced HPV-16 E7-induced VEGF protein secretion (Figure 4B), and the similar results were found at *VEGF* mRNA level (Figure 4C). Taken together, our results indicated that MAOA knockout inhibited HIF-1α/VEGF signaling induced by HPV-16 E7 in NSCLC cells.

To determine whether the degradation of HIF-1α protein was through the 26S proteasome system, A549 and NCI-H460 (16 E7 and 16 E7-MAOA KO) cells were treated with MG-132 (20 mmol/L), a specific 26S proteasome inhibitor. Our results showed that HIF-1α protein accumulation in 16 E7-MAOA KO cells was significantly lower than that in 16 E7 cells (Figure 4D), suggesting that MAOA knockout promoted the degradation of HPV-16 E7-induced HIF-1α protein possibly *via* 26S proteasome pathway in NSCLC cells.

MAOA was reported to stabilize HIF-1α through the increase of ROS production 15. To identify whether HPV-16 E7 oncoprotein enhanced HIF-1α protein accumulation through MAOA/ROS signaling, we detected the level of intracellular ROS in A549 and NCI-H460 cells (empty vector, 16 E7 and 16 E7-MAOA KO). Our results showed that HPV-16 E7 oncoprotein promoted ROS generation, but MAOA knockout significantly reduced HPV-16 E7-induced ROS generation (Figure 4E).

### MAOA played a role in HPV-16 E7-activated ERK1/2 but not AKT signaling pathway

**Figure 5:**
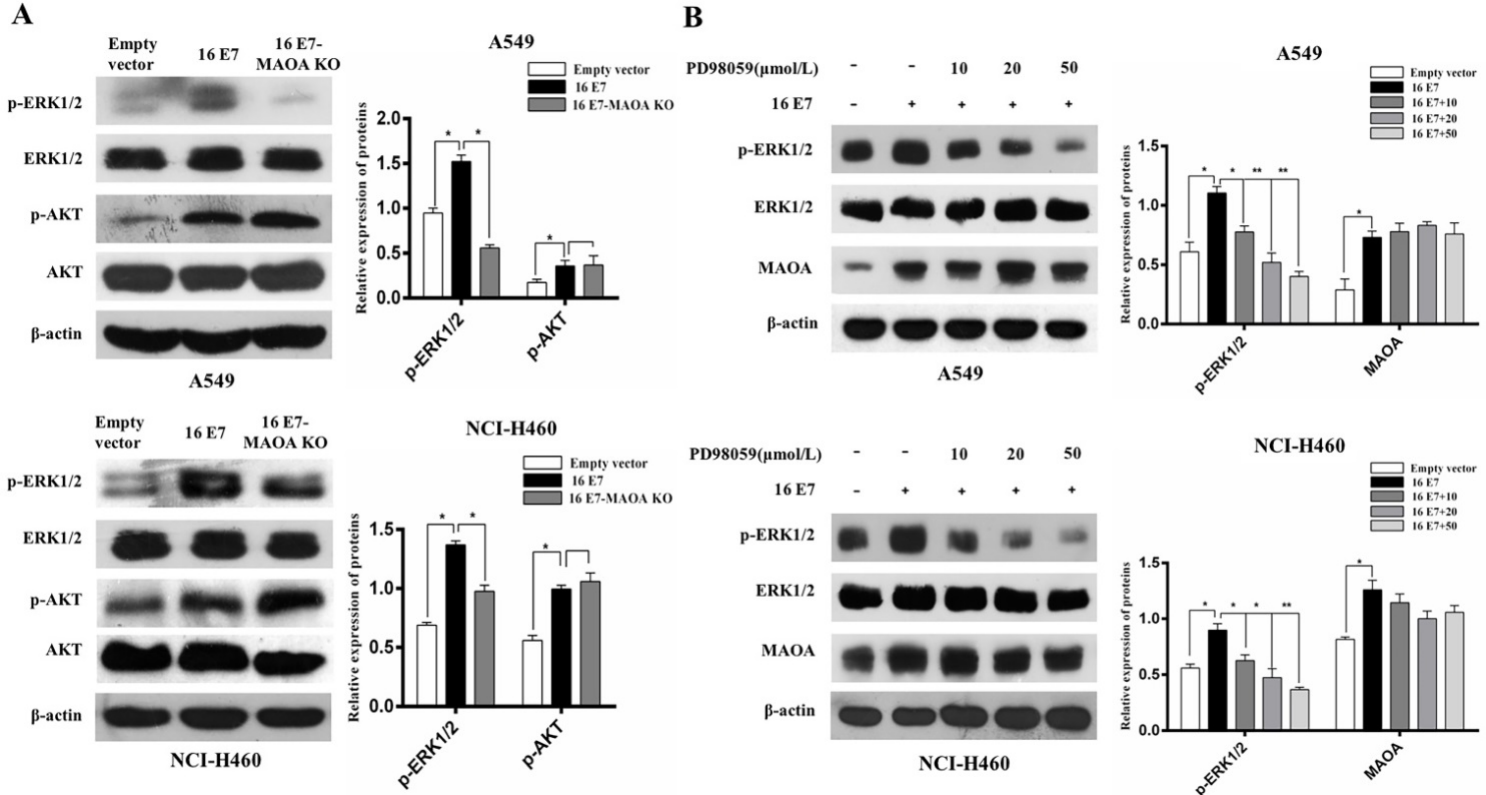
**MAOA knockout inhibited HPV-16 E7-induced activation of ERK1/2 but not AKT in NSCLC cells. (A)** The phosphorylated ERK1/2 and AKT levels in A549 and NCI-H460 cells (Empty vector, 16 E7, and 16 E7-MAOA KO) were determined by Western blotting. **(B)** A549 and NCI-H460 cells (16 E7) were pretreated for 24 h with different concentrations of PD98059, a specific ERK1/2 inhibitor, followed by analysis of MAOA protein expression. All data are expressed as mean±SD of three independent experiments. *P < 0.05, **P < 0.01.

ERK and PI3K/AKT signaling pathways can promote growth, migration, and invasion of NSCLC 41, 42. Our previous studies also demonstrated that HPV-16 E7 oncoprotein promoted the activation of ERK1/2 and PI3K/AKT pathways in A549 and NCI-H460 cells 33, 43. However, the roles of MAOA in HPV-16 E7-induced activation of ERK1/2 and PI3K/AKT pathways remain unknown. Therefore, we examined the effect of MAOA knockout on the activation of ERK1/2 and PI3K/AKT pathways. Our results showed that MAOA knockout significantly inhibited phosphorylated ERK1/2 (p-ERK1/2) expression but did not affect phosphorylated AKT (p-AKT) expression induced by HPV-16 E7 (Figure 5A), indicating MAOA might play a role in HPV-16 E7-induced ERK1/2 but not AKT activation. To further investigate whether ERK1/2 pathway was involved in HPV-16 E7-induced MAOA expression, the stable HPV-16 E7-overexpressing cells (16E7 cells) were treated with different concentrations of PD98059, a specific ERK1/2 inhibitor. The results showed that PD98059 did not affect HPV-16 E7-induced MAOA expression (Figure 5B). Taken together, these results suggested that MAOA might mediate HPV-16 E7-induced ERK1/2 activation, but ERK1/2 signaling pathway was not involved in the expression of MAOA induced by HPV-16 E7 oncoprotein.

### MAOA knockout inhibited HPV-16 E7-induced growth, metastasis, and expression of EMT-related markers and HIF-1α proteins *in vivo*

**Figure 6:**
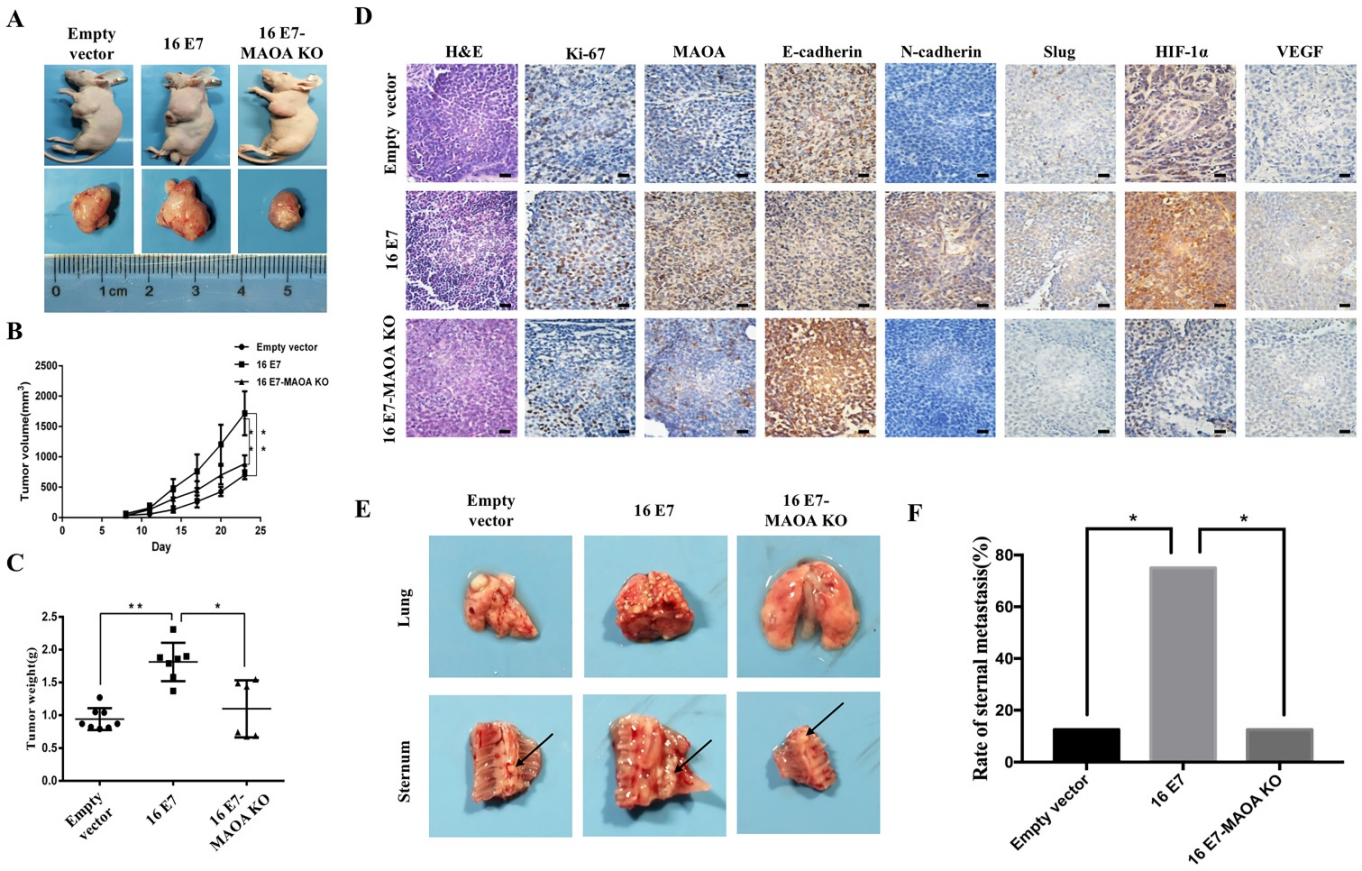
** MAOA knockout inhibited HPV-16 E7-induced NSCLC growth, metastasis, and expression of EMT-related markers and HIF-1α proteins *in vivo*.** The stable NCI-H460 cells (Empty vector, 16 E7, and 16 E7-MAOA KO) were respectively injected into subcutaneous **(A-D)** and intrapulmonary **(E,F)** of nude mice (*n* = 8 mice/group). **(A)** The representative results of tumor growth. **(B)** The volume of subcutaneous xenograft tumors. **(C)** The weight of subcutaneous xenograft tumors. **(D)** Immunohistochemical staining results of Ki-67, MAOA, E-cadherin, N-cadherin, Slug, HIF-1α, and VEGF proteins in subcutaneous xenograft tumor tissues of nude mice. Scale bar =100 µm. **(E)** The sternal metastasis of NCI-H460 intrapulmonary tumors. **(F)** The rate of sternal metastasis. All data are expressed as mean±SD of three independent experiments. *P < 0.05, **P < 0.01.

To further confirm *in vitro* results, we established NCI-H460 subcutaneous xenograft and *in situ* intrapulmonary animal models. In subcutaneous xenograft animal model, the rate of subcutaneous tumorigenesis in the nude mice injected with HPV-16 E7-overexpressing NCI-H460 cells (16 E7 group) was 100%, which was much higher than that in the nude mice injected with HPV-16 E7 overexpression and MAOA knockout cells (16 E7-MAOA KO group, 75%). The tumor volume and weight in 16 E7 group were significantly increased as compared with empty vector and 16 E7-MAOA KO groups (Figure 6A-C). Moreover, immunohistochemistry results showed that HPV-16 E7 enhanced the expression of Ki67, a proliferation-related protein, but MAOA knockout inhibited HPV-16 E7-enhanced Ki67 expression in subcutaneous xenograft tumors (Figure 6D). Furthermore, *in situ* intrapulmonary mouse model, HPV-16 E7 was found to promote sternal metastasis, but MAOA knockout dramatically inhibited HPV-16 E7-induced sternal metastasis (*P*<0.05, Figure 6E,F). Taken together, these results indicated that MAOA might play a role in the growth and metastasis of NCI-H460 NSCLC.

To further verify the effects of MAOA on HPV-16 E7-induced EMT and HIF-1α expression *in vivo*, the expression of EMT markers and HIF-1α in tumor tissues was analyzed by immunohistochemistry. We found that 16 E7-MAOA KO group showed the stronger expression of E-cadherin and the weaker expression of N-cadherin and Slug as compared with 16E7 group in subcutaneous xenograft tumors (Figure 6D). These results suggested that MAOA knockout could inhibit HPV-16 E7-induced EMT, which was consistent with the data *in vitro*. Moreover, we further demonstrated that MAOA knockout inhibited HPV-16 E7-induced HIF-1α and VEGF protein expression in subcutaneous xenograft tumors (Figure 6D). Collectively, the* in vivo* results further confirmed that MAOA might play a role in HPV-16 E7-induced EMT and HIF-1α/VEGF expression in NSCLC cells.

## Discussion

A growing body of evidence has demonstrated that MAOA plays a role in the progression of various cancers including NSCLC, and the expression of MAOA in various cancers is different 15, 18-22. MAOA was reported to highly express in prostate cancer, classical hodgkin's lymphoma, and breast cancer tissues, but a lower expression of MAOA was found in liver cancer, pancreatic ductal adenocarcinoma, and cholangiocarcinoma 15, 18-22. In prostate cancer, MAOA induced EMT and stabilized the expression of HIF-1α protein, ultimately promoting the growth, invasion, and metastasis 15. Bioinformatics analysis found that MAOA might be a potential target of diagnosis and treatment for lung cancer 44. Our previous study demonstrated that MAOA was highly expressed in NSCLC and correlated with EMT, suggesting that MAOA might play a role in promoting NSCLC progression by regulating EMT 23.

EMT is essential for promoting cancer metastasis 45. Our previous studies demonstrated that HPV-16 E7 oncoprotein up-regulated the expression of EMT-related markers including N-cadherin, Vimentin, ZEB1, and Snail1 and down-regulated the expression of E-cadherin and ZO-1 in A549 and NCI-H460 NSCLC cells 12. Similarly, Wu et al 15 also reported that MAOA induced EMT, thereby promoting prostate cancer growth, invasion, and metastasis. Additionally, it was reported that MAOA might promote the metastatic potential of lung cancer cells 46. In this study, we demonstrated that HPV-16 E7 oncoprotein enhanced MAOA expression at both protein and mRNA levels (Figure 1C,D), and MAOA knockout inhibited the migration, invasion, and EMT induced by HPV-16 E7 oncoprotein in A549 and NCI-H460 NSCLC cells (Figure 2,3). These results support the essential role of MAOA in the regulation of EMT induced by HPV-16 E7 in NSCLC cells.

Previous studies demonstrated that hypoxic microenvironment plays a key role in cancer progression and metastasis, and HIF-1α overexpression was confirmed in many solid tumors 47, 48. Moreover, HIF-1α was found to mediate VEGF expression in tumor microenvironment 40. Our previous study found that HPV-16 E7 oncoprotein promoted HIF-1α protein accumulation and HIF-1α-dependent VEGF expression in NSCLC cells 36, 49. In the present study, we further found that HPV-16 E7-induced HIF-1α protein accumulation and HIF-1α-dependent VEGF expression were inhibited by MAOA knockout, and MAOA knockout enhanced the degradation of HPV-16 E7-induced HIF-1α protein possibly *via* 26S proteasome pathway in NSCLC cells (Figure 4A-D). Recently, in prostate cancer, MAOA was reported to stabilize HIF-1α and mediate hypoxia by increasing ROS that can repress PHD activity, thereby enhancing the growth, invasion, and metastasis of prostate cancer cells 15. Interestingly, in this study, we firstly demonstrated HPV-16 E7 oncoprotein up-regulated ROS level, but MAOA knockout inhibited this effect in NSCLC cells (Figure 4E). Taken together, these results suggest that MAOA may be involved in the stabilization of HPV-16 E7-induced HIF-1α protein by regulating ROS level in NSCLC cells.

ERK1/2 and AKT activation was reported to be associated with malignant transformation 50. Moreover, our previous studies found that HPV-16 E7 oncoprotein promoted the activation of ERK1/2 and AKT in A549 and NCI-H460 NSCLC cells 43, 49. In this study, we demonstrated that MAOA knockout inhibited HPV-16 E7-induced activation of ERK1/2 but not AKT, indicating that MAOA is essential in HPV-16 E7-induced ERK1/2 activation (Figure 5A). However, in turn, we found that PD98059, a specific ERK1/2 inhibitor, had no obvious effect on HPV-16 E7-induced MAOA expression (Figure 5B). Furthermore, our previous study showed that ERK1/2 signaling pathway was not involved in HPV-16 E7-induced HIF-1α protein accumulation 43. Therefore, our previous and present studies suggest that MAOA may be as an upstream regulator to mediate HPV-16 E7-induced ERK1/2 activation, but ERK1/2 signaling has no feedback effect on HPV-16 E7-induced MAOA and HIF-1α expression. However, previous studies demonstrated that HIF-1α was closely related to the cell proliferation and invasion abilities of lung cancer cells, which was associated with the regulation of ERK1/2 pathway, and ERK1/2 phosphorylation increased HIF-1α protein expression 51-53. The difference between our results and previous studies in the relationship of ERK1/2 phosphorylation and HIF-1α protein accumulation may be due to the effect of MAOA/ROS/PHD/HIF-1α pathway. Our result showed that HPV-16 E7 oncoprotein up-regulated ROS level through MAOA (Figure 4E), and MAOA was demonstrated to stabilize HIF-1α through ROS/PHD pathway 15. Therefore, these results suggest that HPV-16 E7 oncoprotein can still accumulate HIF-1α protein through MAOA/ROS/PHD/HIF-1α pathway, even if ERK1/2 signaling pathway is suppressed, which needs to be further studied.

In order to confirm the results *in vitro*, NCI-H460 cells (Empty vector, 16 E7, and 16 E7-MAOA KO) were injected into subcutaneous and intrapulmonary of nude mice to construct tumor models. We found that the knockout of MAOA significantly inhibited HPV-16 E7-induced NCI-H460 NSCLC growth and metastasis. Moreover, the *in vivo* results also verified that the knockout of MAOA inhibited HPV-16 E7-induced EMT and HIF-1α protein expression (Figure 6). Collectively, both *in vitro* and *in vivo* results suggest that MAOA may play a role in HPV-16 E7-induced EMT and HIF-1α protein expression.

**Figure 7:**
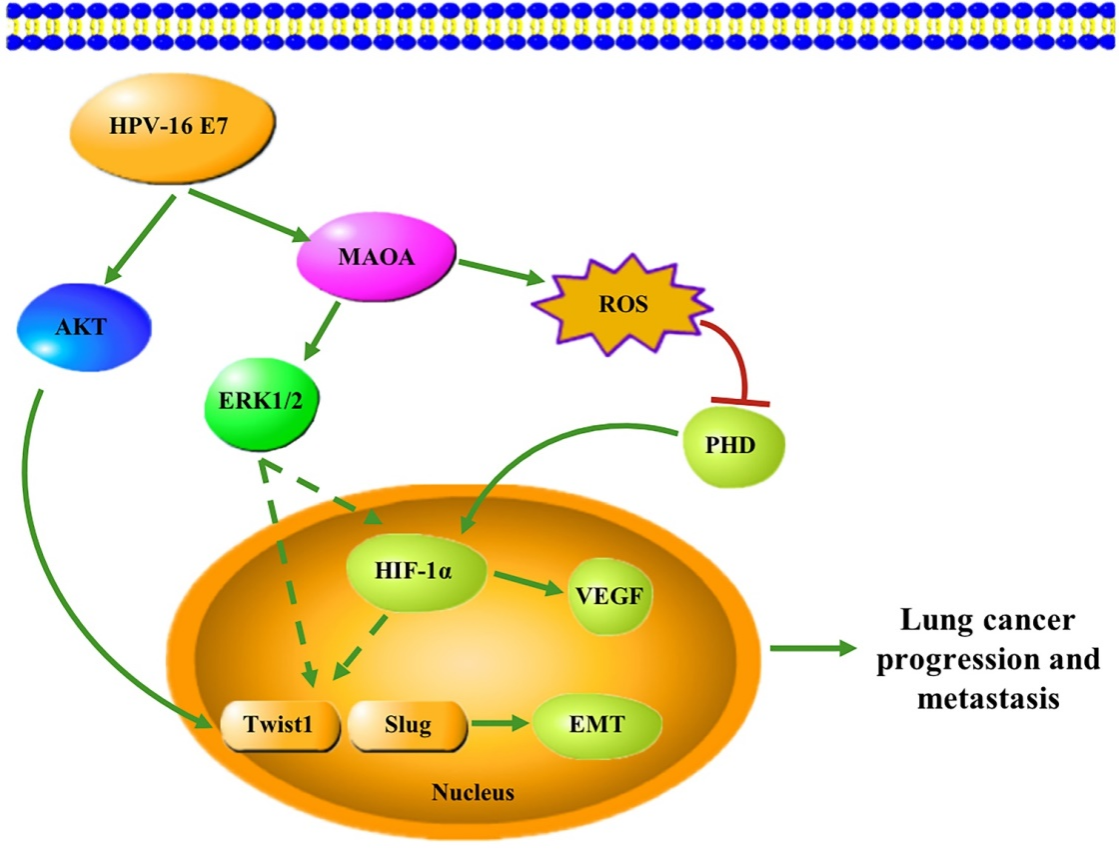
A proposed working model for the role of MAOA in HPV-16 E7-induced EMT and HIF-1α protein accumulation in NSCLC cells.

In conclusion, in this study, we firstly demonstrated that HPV-16 E7 oncoprotein enhanced MAOA expression and MAOA played a key role in HPV-16 E7-induced EMT and HIF-1α protein accumulation in A549 and NCI-H460 NSCLC cells (Figure **7**). These findings suggest that MAOA may be a potential target for the prevention and treatment of HPV-related NSCLC.

## Abbreviations

HPV: human papillomavirus
EMT: epithelial-mesenchymal transition
HIF-1α: hypoxia-inducible factor-1α
NSCLC: non-small cell lung cancer
MAOA: monoamine oxidases A
EMT-TFs: EMT-related transcription factors
ROS: reactive oxygen species
PHD: prolyl hydroxylase enzymes
16 E7: the stable HPV-16 E7-overexpressing A549 and NCI-H460 cell lines
16 E7-MAOA KO: the stable cell lines with HPV-16 E7 overexpression and MAOA knockout

## References

[1] Bray F, Ferlay J, Soerjomataram I, Siegel RL, Torre LA, Jemal A (2018). Global cancer statistics 2018: GLOBOCAN estimates of incidence and mortality worldwide for 36 cancers in 185 countries. CA Cancer J Clin.

[2] Tang ER, Schreiner AM, Pua BB (2014). Advances in lung adenocarcinoma classification: a summary of the new international multidisciplinary classification system (IASLC/ATS/ERS). J Thorac Dis.

[3] Gardiner N, Jogai S, Wallis A (2014). The revised lung adenocarcinoma classification-an imaging guide. J Thorac Dis.

[4] Pikin OV, Riabov AB, Trakhtenberg AK, Glushko VA, Kolbanov KI, Amiraliev AM (2016). [Analysis of postoperative complications after pneumo-n-ectomy using thoracic morbidity and mortality (tmm) system in nsclc patients for a 5-year period]. Khirurgiia (Mosk).

[5] Svaton M, Fiala O, Pesek M, Bortlicek Z, Minarik M, Benesova L (2016). The Prognostic Role of KRAS Mutation in Patients with Advanced NSCLC Treated with Second- or Third-line Chemotherapy. Anticancer Res.

[6] Zhang EY, Tang XD (2012). Human papillomavirus type 16/18 oncoproteins: potential therapeutic targets in non-smoking associated lung cancer. Asian Pac J Cancer Prev.

[7] de Freitas AC, Gurgel AP, de Lima EG, de Franca Sao Marcos B, do Amaral CM (2016). Human papillomavirus and lung cancinogenesis: an overview. J Cancer Res Clin Oncol.

[8] Lin FC, Huang JY, Tsai Sc, Nfor On, Chou Mc, Wu Mf (2016). The association between human papillomavirus infection and female lung cancer: A population-based cohort study. Medicine (Baltimore).

[9] Robinson LA, Jaing CJ, Pierce Campbell C, Magliocco A, Xiong Y, Magliocco G (2016). Molecular evidence of viral DNA in non-small cell lung cancer and non-neoplastic lung. Br J Cancer.

[10] Syrjanen KJ (1979). Condylomatous changes in neoplastic bronchial epithelium. Report of a case. Respiration.

[11] Zhai K, Ding J, Shi HZ (2015). HPV and lung cancer risk: a meta-analysis. J Clin Virol.

[12] Zhang W, Wu X, Hu L, Ma Y, Xiu Z, Huang B (2017). Overexpression of Human Papillomavirus Type 16 Oncoproteins Enhances Epithelial-Mesenchymal Transition via STAT3 Signaling Pathway in Non-Small Cell Lung Cancer Cells. Oncol Res.

[13] Wang X, Zhang Z, Cao H, Niu W, Li M, Xi X (2017). Human papillomavirus type 16 E6 oncoprotein promotes proliferation and invasion of non-small cell lung cancer cells through Toll-like receptor 3 signaling pathway. J Med Virol.

[14] de Oliveira THA, do Amaral CM, de Franca Sao Marcos B, Nascimento KCG, de Miranda Rios AC, Quixabeira DCA (2018). Presence and activity of HPV in primary lung cancer. J Cancer Res Clin Oncol.

[15] Wu J, Shao C, Li X, Li Q, Hu P, Shi C (2014). Monoamine oxidase A mediates prostate tumorigenesis and cancer metastasis. J Clin Invest.

[16] Rozycka A, Slopien R, Slopien A, Dorszewska J, Seremak-Mrozikiewicz A, Lianeri M (2016). The MAOA, COMT, MTHFR and ESR1 gene polymorphisms are associated with the risk of depression in menopausal women. Maturitas.

[17] Liu Z, Huang L, Luo XJ, Wu L, Li M (2016). MAOA Variants and Genetic Susceptibility to Major Psychiatric Disorders. Mol Neurobiol.

[18] Huang L, Frampton G, Rao A, Ks Z, W C, Jm L (2012). Monoamine oxidase A expression is suppressed in human cholangiocarcinoma via coordinated epigenetic and IL-6-driven events. Lab Invest.

[19] Li J, Yang XM, Wang YH, Feng MX, Liu XJ, Zhang YL (2014). Monoamine oxidase A suppresses hepatocellular carcinoma metastasis by inhibiting the adrenergic system and its transactivation of EGFR signaling. J Hepatol.

[20] Li P, Siddiqi IN, Mottok A, Loo EY, Wu CH, Cozen W (2017). Monoamine oxidase A is highly expressed in classical Hodgkin lymphoma. J Pathol.

[21] Satram-Maharaj T, Nyarko JN, Kuski K, Fehr K, Pennington PR, Truitt L (2014). The monoamine oxidase-A inhibitor clorgyline promotes a mesenchymal-to-epithelial transition in the MDA-MB-231 breast cancer cell line. Cell Signal.

[22] Jiang SH, Li J, Dong FY, Yang JY, Liu DJ, Yang XM (2017). Increased Serotonin Signaling Contributes to the Warburg Effect in Pancreatic Tumor Cells Under Metabolic Stress and Promotes Growth of Pancreatic Tumors in Mice. Gastroenterology.

[23] Liu F, Hu L, Ma Y, Huang B, Xiu Z, Zhang P (2018). Increased expression of monoamine oxidase A is associated with epithelial to mesenchymal transition and clinicopathological features in non-small cell lung cancer. Oncol Lett.

[24] Lin PL, Cheng YM, Wu DW, Huang YJ, Lin HC, Chen CY (2017). A combination of anti-PD-L1 mAb plus Lm-LLO-E6 vaccine efficiently suppresses tumor growth and metastasis in HPV-infected cancers. Cancer Med.

[25] He W, Sun Z, Liu Z (2015). Silencing of TGM2 reverses epithelial to mesenchymal transition and modulates the chemosensitivity of breast cancer to docetaxel. Exp Ther Med.

[26] Da C, Liu Y, Zhan Y, Liu K, Wang R (2016). Nobiletin inhibits epithelial-mesenchymal transition of human non-small cell lung cancer cells by antagonizing the TGF-beta1/Smad3 signaling pathway. Oncol Rep.

[27] Kurimoto R, Iwasawa S, Ebata T, Ishiwata T, Sekine I, Tada Y (2016). Drug resistance originating from a TGF-beta/FGF-2-driven epithelial-to-mesenchymal transition and its reversion in human lung adenocarcinoma cell lines harboring an EGFR mutation. Int J Oncol.

[28] Mezencev R, Matyunina LV, Jabbari N, McDonald JF (2016). Snail-induced epithelial-to-mesenchymal transition of MCF-7 breast cancer cells: systems analysis of molecular changes and their effect on radiation and drug sensitivity. BMC Cancer.

[29] Zhou JP, Gao ZL, Zhou ML, He MY, Xu XH, Tao DT (2015). Snail interacts with Id2 in the regulation of TNF-alpha-induced cancer cell invasion and migration in OSCC. Am J Cancer Res.

[30] Yu S, Yan C, Yang X, He S, Liu J, Qin C (2016). Pharmacoproteomic analysis reveals that metapristone (RU486 metabolite) intervenes E-cadherin and vimentin to realize cancer metastasis chemoprevention. Sci Rep.

[31] Yue QY, Zhang Y (2018). Effects of Linc00460 on cell migration and invasion through regulating epithelial-mesenchymal transition (EMT) in non-small cell lung cancer. Eur Rev Med Pharmacol Sci.

[32] Eo J, Lee HE, Nam GH, Kwon YJ, Choi Y, Choi BH (2016). Association of DNA methylation and monoamine oxidase A gene expression in the brains of different dog breeds. Gene.

[33] Liu J, Huang B, Xiu Z, Zhou Z, Liu J, Li X (2018). PI3K/Akt/HIF-1alpha signaling pathway mediates HPV-16 oncoprotein-induced expression of EMT-related transcription factors in non-small cell lung cancer cells. J Cancer.

[34] Rankin EB, Giaccia AJ (2016). Hypoxic control of metastasis. Science.

[35] Zhang J, Wu Y, Lin YH, Guo S, Ning PF, Zheng ZC (2018). Prognostic value of hypoxia-inducible factor-1 alpha and prolyl 4-hydroxylase beta polypeptide overexpression in gastric cancer. World J Gastroenterol.

[36] Li G, He L, Zhang E, Shi J, Zhang Q, Le AD (2011). Overexpression of human papillomavirus (HPV) type 16 oncoproteins promotes angiogenesis via enhancing HIF-1alpha and VEGF expression in non-small cell lung cancer cells. Cancer Lett.

[37] Ma Y, Wu X, Xiu Z, Liu X, Huang B, Hu L (2018). Cytochalasin H isolated from mangrove-derived endophytic fungus induces apoptosis and inhibits migration in lung cancer cells. Oncol Rep.

[38] Shiau MY, Fan L, Yang S, Tsao C, Lee H, Cheng Y (2013). Human papillomavirus up-regulates MMP-2 and MMP-9 expression and activity by inducing interleukin-8 in lung adenocarcinomas. PLoS One.

[39] Meng X, Ezzati P, Wilkins JA (2011). Requirement of podocalyxin in TGF-beta induced epithelial mesenchymal transition. PLoS One.

[40] De Francesco EM, Sims AH, Maggiolini M, Sotgia F, Lisanti MP, Clarke RB (2017). GPER mediates the angiocrine actions induced by IGF1 through the HIF-1alpha/VEGF pathway in the breast tumor microenvironment. Breast Cancer Res.

[41] Wei CH, Wu G, Cai Q, Gao XC, Tong F, Zhou R (2017). MicroRNA-330-3p promotes cell invasion and metastasis in non-small cell lung cancer through GRIA3 by activating MAPK/ERK signaling pathway. J Hematol Oncol.

[42] Jiang M, Zhou LY, Xu N, An Q (2019). Hydroxysafflor yellow A inhibited lipopolysaccharide-induced non-small cell lung cancer cell proliferation, migration, and invasion by suppressing the PI3K/AKT/mTOR and ERK/MAPK signaling pathways. Thorac Cancer.

[43] Liu F, Lin B, Liu X, Zhang W, Zhang E, Hu L (2016). ERK Signaling Pathway Is Involved in HPV-16 E6 but not E7 Oncoprotein-Induced HIF-1alpha Protein Accumulation in NSCLC Cells. Oncol Res.

[44] Li J, Yu H, Ma Y, Zhao M, Tang J (2017). Identification of genes associated with lung cancer by bioinformatics analysis. Eur Rev Med Pharmacol Sci.

[45] Tsai IT, Kuo CC, Liou JP, Chang JY (2018). Novel microtubule inhibitor MPT0B098 inhibits hypoxia-induced epithelial-to-mesenchymal transition in head and neck squamous cell carcinoma. J Biomed Sci.

[46] Dhabal S, Das P, Biswas P, Kumari P, Yakubenko VP, Kundu S (2018). Regulation of monoamine oxidase A (MAO-A) expression, activity, and function in IL-13-stimulated monocytes and A549 lung carcinoma cells. J Biol Chem.

[47] Liu ZJ, Semenza G, Zhang HF (2015). Hypoxia-inducible factor 1 and breast cancer metastasis. J Zhejiang Univ Sci B.

[48] Fan R, Hou WJ, Zhao YJ, Liu SL, Qiu XS, Wang EH (2016). Overexpression of HPV16 E6/E7 mediated HIF-1alpha upregulation of GLUT1 expression in lung cancer cells. Tumour Biol.

[49] Zhang E, Feng X, Liu F, Zhang P, Liang J, Tang X (2014). Roles of PI3K/Akt and c-Jun signaling pathways in human papillomavirus type 16 oncoprotein-induced HIF-1alpha, VEGF, and IL-8 expression and in vitro angiogenesis in non-small cell lung cancer cells. PLoS One.

[50] Tian W, Zhang H, Zhang Y, Wang Y, Zhang Y, Xue F (2020). High level of visfatin and the activation of Akt and ERK1/2 signaling pathways are associated with endometrium malignant transformation in polycystic ovary syndrome. Gynecol Endocrinol.

[51] Cheng CW, Chen PM, Hsieh YH, Weng CC, Chang CW, Yao CC (2015). Foxo3a-mediated overexpression of microRNA-622 suppresses tumor metastasis by repressing hypoxia-inducible factor-1α in ERK-responsive lung cancer. Oncotarget.

[52] Jang BC (2012). The fruit juice of Morinda citrifolia (noni) downregulates HIF-1α protein expression through inhibition of PKB, ERK-1/2, JNK-1 and S6 in manganese-stimulated A549 human lung cancer cells. Int J Mol Med.

[53] Qian J, Bai H, Gao Z, Dong YU, Pei J, Ma M (2016). Downregulation of HIF-1α inhibits the proliferation and invasion of non-small cell lung cancer NCI-H157 cells. Oncol Lett.

